# Pre‐Weaning Administration of a PGF2α Analogue (Cloprostenol) and Its Influence on Early Reproductive Indicators in Sows Under Commercial Farm Conditions

**DOI:** 10.1111/rda.70290

**Published:** 2026-09-30

**Authors:** Sara Crespo, Joaquín Gadea

**Affiliations:** ^1^ CEFUSA Murcia Spain; ^2^ Department of Physiology University of Murcia, International Excellence Campus for Higher Education and Research “Campus Mare Nostrum” and Biomedical Research Institute of Murcia Pascual Parrilla–IMIB Murcia Spain

## Abstract

Efficient reproductive management in commercial swine production requires minimising non‐productive days and ensuring rapid return to oestrus after weaning. The present study evaluated the effect of pre‐weaning administration of cloprostenol, a PGF2α analogue, on early reproductive indicators in sows under commercial farm conditions. A total of 734 clinically healthy multiparous sows (parity 2–9) from a commercial farm were randomly allocated to two groups before weaning. Control sows (*n* = 367) received no treatment, whereas treated sows (*n* = 367) received an intramuscular injection of cloprostenol sodium 12 h before piglet removal. Reproductive parameters evaluated included the weaning‐to‐first‐service interval (WFSI), proportion of sows in oestrus within 7 days after weaning, pregnancy rate, early endometritis and abortion rate within the first 42 days of gestation. Sows treated with cloprostenol showed a significantly shorter WFSI compared with control sows (treated: 4.94 ± 0.18 days vs. control: 6.41 ± 0.27 days; *p* = 0.001) and a higher proportion of oestrus within 7 days after weaning (93.19% vs. 84.2%; *p* = 0.008). Pregnancy rate and early endometritis incidence were not affected by treatment. However, cloprostenol significantly reduced abortion rate during early gestation (0.55% vs. 3.27%; *p* = 0.006). Under the conditions of this field study, pre‐weaning administration of cloprostenol was associated with a shorter WFSI, a higher proportion of sows showing oestrus within 7 days after weaning, and a lower early abortion rate. These findings suggest that cloprostenol may influence early reproductive indicators in multiparous sows under commercial farm conditions, although further studies including endocrine, ovarian and farrowing outcomes are required.

## 1 Introduction

Efficient reproductive management in commercial swine production requires minimising non‐productive days and ensuring timely and promoting a rapid return to oestrus after weaning. The weaning to first service interval is therefore an important indicator of herd reproductive efficiency. Prostaglandin F2α (PGF2α) and its analogues have uterotonic and luteolytic properties, although their reproductive effects in sows have been variable depending on the timing of administration and the physiological status of the animals (De Rensis et al. 2012; Peña et al. 2001). PGF2α analogues have luteolytic activity when corpora lutea are responsive; however, the early porcine corpus luteum is refractory to a single exogenous PGF2α treatment until approximately Day 12 of the oestrous cycle (De Rensis et al. 2012; Diaz and Wiltbank 2004).

In commercial sow herds, multiparous sows often display greater variability in uterine involution and ovarian activity at weaning compared with younger parity groups, potentially resulting in extended weaning‐to‐oestrus intervals and suboptimal reproductive outcomes. Furthermore, suboptimal uterine clearance or residual luteal activity around weaning may contribute to delayed post‐weaning oestrus expression and early reproductive inefficiency in a subset of sows. The peri‐weaning interval thus represents a critical physiological window during which endocrine reactivation and uterine remodelling occur concomitantly, and targeted interventions during this period may influence early reproductive success.

Although most sows undergo complete luteal regression after parturition and remain in a state of lactational anoestrus until weaning, several studies have reported considerable variation in ovarian activity during lactation. Elevated progesterone concentrations at weaning have been detected in a proportion of sows 15 out of 191 (7.8%) showed values > 3 ng/mL (Elbers et al. 1994), suggesting the presence of functional luteal tissue or recent ovulatory events. In addition, lactational ovulation has been reported under commercial conditions, particularly in hyperprolific multiparous sows exposed to management factors such as intermittent suckling, piglet cross‐fostering, or reduced suckling‐induced inhibition of gonadotropin secretion (van Wettere et al. 2017; Weaver et al. 2024). Consequently, a subset of sows may reach weaning with residual luteal activity capable of delaying the endocrine transition required for a rapid return to oestrus.

Given these physiological considerations, administration of a PGF2α analogue shortly before weaning may theoretically influence early post‐weaning reproductive events through its uterotonic action and, in selected animals, through luteolysis of responsive luteal tissue. Its uterotonic properties may support myometrial activity and physiological uterine clearance during the peri‐weaning period, although this should not be interpreted as treatment of established uterine inflammation. These early reproductive indicators include the weaning‐to‐first‐service interval, oestrus expression within 7 days after weaning, pregnancy establishment and early gestational losses.

The objective of this study was to evaluate the effect of administering cloprostenol sodium, a PGF2α analogue, 12 h before weaning on early reproductive indicators up to 42 days of gestation in multiparous sows under commercial farm conditions.

## 2 Material and Methods

### 2.1 Experimental Design

A total of 734 clinically healthy multiparous sows (parity 2–9) from a commercial farm located in southeastern Spain were enrolled in the study. The farm had approximately 1000 breeding sows and an average production rhythm of approximately 45 farrowings per week. Mean lactation length was 28 days, and mean piglet weight at weaning was approximately 6 kg. The study included 16 consecutive weekly weaning batches. Animals were randomly allocated before weaning into one of two experimental groups, ensuring a balanced parity distribution between treatments. The control group (*n* = 367) received no prostaglandin treatment, whereas the PGF2α group (*n* = 367) received a single intramuscular injection of 2 mL cloprostenol sodium solution (Planate, 87.50 μg/mL; MSD Animal Health España), equivalent to 175 μg cloprostenol per sow, administered 12 h before piglet removal.

Following weaning, oestrus detection was performed twice daily using boar exposure. Sows detected in standing oestrus were inseminated by post‐cervical artificial insemination at 0 and 24 h after the onset of oestrus, according to standard farm reproductive management protocols.

Individual reproductive data were systematically recorded and subsequently analysed. The evaluated variables included the weaning‐to‐first‐service interval (WFSI), pregnancy rate confirmed by ultrasonography, incidence of purulent endometritis and abortion rate within the first 42 days of gestation.

No endocrine evaluations were performed before weaning. Serum progesterone concentrations were not measured and ovarian ultrasonographic examinations were not conducted to assess the presence of functional corpora lutea or other ovarian structures. Therefore, ovarian status at the time of treatment administration was not determined in the present field study.

Endometritis was diagnosed during routine post‐service reproductive monitoring based on the presence of purulent or mucopurulent vulvar discharge detected during clinical examinations. All evaluations were performed by the same trained farm personnel according to standard herd health procedures.

### 2.2 Ethical Issues

All procedures complied with national and institutional guidelines for animal care and use. The study involved standard farm management procedures and did not require additional ethical approval according to institutional guidelines.

### 2.3 Statistical Analysis

Normality of continuous variables was assessed using the Kolmogorov–Smirnov and Shapiro–Wilk tests, which indicated a significant deviation from normality (*p* < 0.05). Therefore, group comparisons for continuous variables were performed using the Mann–Whitney *U* test.

Binary outcomes, including pregnancy, early endometritis and abortion rates were analysed using logistic regression models. Treatment (cloprostenol vs. control), parity, WFSI and lactation duration were included as independent variables. Parity was included as a continuous variable. Odds ratios (OR) with 95% confidence intervals (CI) were calculated. Model fit was assessed using the Hosmer–Lemeshow goodness‐of‐fit test, and statistical significance was set at *p* < 0.05. All analyses were performed using IBM SPSS Statistics (version 28, IBM Corp., Armonk, NY, USA).

## 3 Results

Sows treated with cloprostenol 12 h before weaning had a significantly shorter weaning‐to‐first‐service interval (WFSI) than control sows (treated: 4.94 ± 0.18 days vs. control: 6.41 ± 0.27 days; *p* = 0.001; Figure 1). The proportion of sows showing oestrus within 7 days after weaning was also higher in the treated group than in the control group (treated: 93.19% vs. control: 84.2%; *p* = 0.008).

**FIGURE 1:**
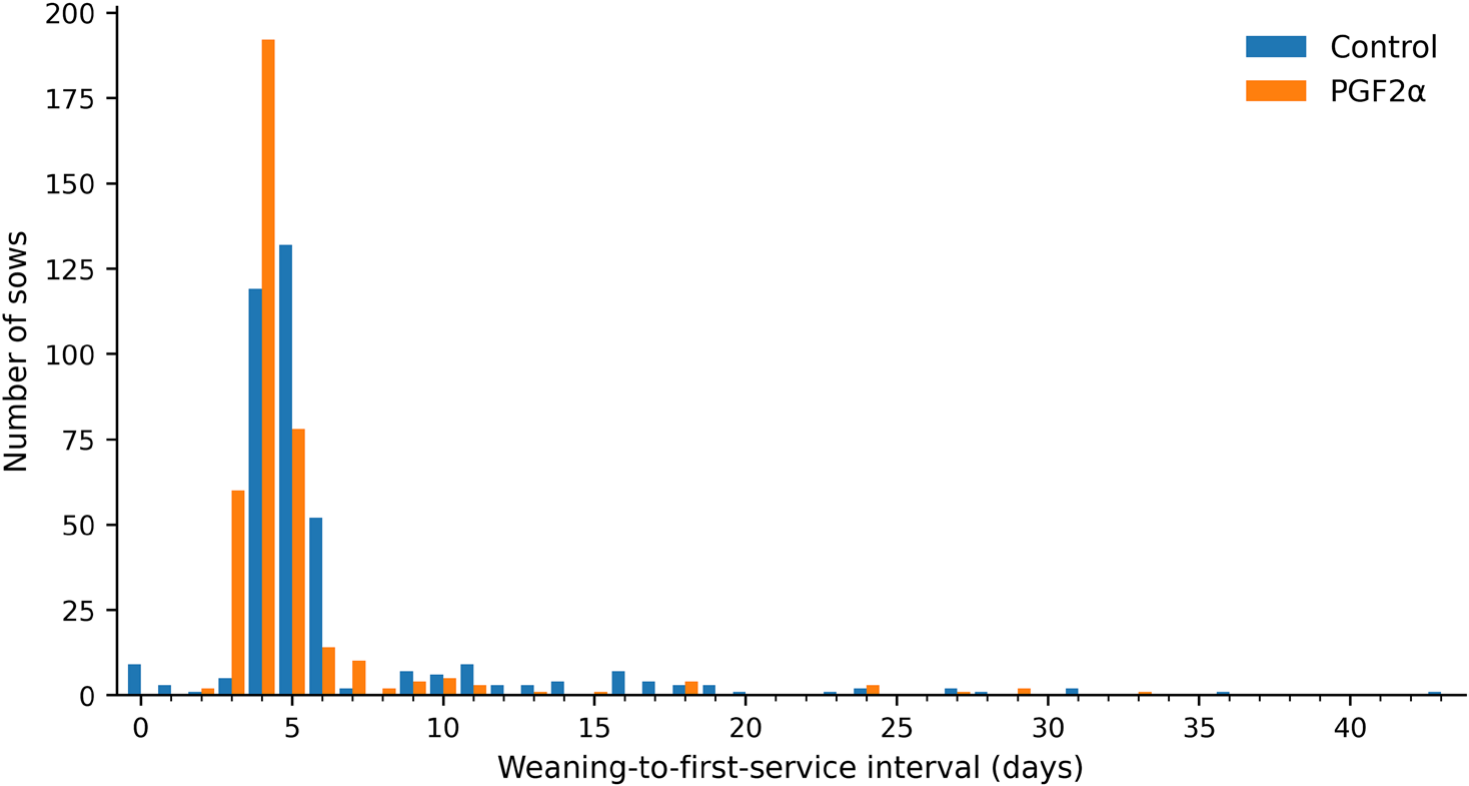
Frequency distribution of the weaning‐to‐first‐service interval (WFSI, days) in control sows and sows treated with cloprostenol 12 h before weaning. Treated sows had a significantly shorter WFSI than control sows (estimated marginal mean ± SD, treated: 4.94 ± 0.18 days vs. control: 6.41 ± 0.27 days; *p* = 0.001).

Binary logistic regression analyses showed that pre‐weaning administration of cloprostenol did not significantly affect pregnancy rate (treated: 93.19% vs. control: 91.55%; *p* > 0.05) or the incidence of early endometritis (treated: 2.72% vs. control: 3.54%; *p* > 0.05). Pregnancy rate was associated with parity (OR = 1.23, 95% CI: 1.07–1.41; *p* = 0.003).

The abortion rate within the first 42 days of gestation was lower in treated sows than in control sows (treated: 0.55% vs. control: 3.27%). In the logistic regression model, cloprostenol treatment was associated with a lower probability of abortion (OR = 0.12, 95% CI: 0.03–0.54; *p* = 0.006). WFSI was also associated with abortion rate (OR = 0.67, 95% CI: 0.48–0.94; *p* = 0.017).

## 4 Discussion

The present field study evaluated the association between pre‐weaning administration of cloprostenol and early reproductive indicators in multiparous sows under commercial farm conditions. Treated sows showed a shorter WFSI and a higher proportion of oestrus expression within 7 days after weaning than control sows. In contrast, pregnancy rate and early endometritis incidence were not significantly affected by treatment. A lower abortion rate within the first 42 days of gestation was observed in treated sows.

The reduction in WFSI is consistent with previous field observations by Peña et al. (2001), who reported a higher proportion of sows showing oestrus within 7 days after d‐cloprostenol treatment during the low‐fertility summer season. However, comparison with that study should be made cautiously, because Peña et al. administered d‐cloprostenol both at weaning and at insemination, used a vulvomucosal route (Peña et al. 2001). In contrast, the present study used a single intramuscular treatment 12 h before weaning. Therefore, the present results should not be interpreted as direct confirmation of the same protocol, but rather as additional field evidence that PGF2α analogues may influence early post‐weaning reproductive events under specific commercial conditions.

The biological basis for the shorter WFSI remains uncertain. PGF2α is involved in several reproductive processes in female swine, including luteal function, uterine contractility, ovulation and embryo attachment (De Rensis et al. 2012). However, a single exogenous PGF2α treatment does not induce complete luteolysis before approximately Day 12 of the oestrous cycle; therefore, cloprostenol should not be considered a general oestrus‐synchronisation tool in sows (De Rensis et al. 2012; Diaz and Wiltbank 2004). A more plausible interpretation is that treatment may have acted in a subset of animals with residual luteal activity or recent ovulatory events around weaning. Lactational oestrus or intralactational ovulation has been reported in sows and may be favoured by management factors that alter suckling stimulus, such as piglet cross‐fostering, intermittent suckling or reduced suckling pressure (van Wettere et al. 2017; Weaver et al. 2024). In such animals, cloprostenol‐induced luteolysis could contribute to a more rapid endocrine transition after weaning.

The possible uterine contribution should also be interpreted cautiously. PGF2α has uterotonic properties and may stimulate myometrial contractions and uterine evacuation of residual material after parturition (De Rensis et al. 2012). However, this should not be interpreted as treatment of established uterine inflammation. In the present study, the incidence of clinically detected endometritis was low and was not significantly affected by treatment, suggesting that any potential uterine effect was not reflected in a measurable reduction in purulent or mucopurulent discharge under the conditions of this herd.

Pregnancy rate was not significantly affected by treatment. This may be explained, at least in part, by the high reproductive performance of the herd, with pregnancy rates above 90% in both groups, leaving limited room for improvement. Similar context‐dependent responses have been reported for prostaglandin‐based interventions in sows, with more evident effects when baseline fertility is low or under seasonal fertility challenges (De Rensis et al. 2012; Kirkwood et al. 2007; Peña et al. 2001). Parity was associated with pregnancy rate in the present study, confirming that sow reproductive status remains an important determinant of early reproductive outcome independently of cloprostenol treatment.

The lower abortion rate observed in treated sows is an interesting finding, but it should be interpreted with caution. Early gestational loss is multifactorial and may be influenced by uterine environment, endocrine status, insemination timing, parity, season and management factors (Christianson 1992). Although improved physiological uterine clearance or a more synchronised endocrine transition could provide plausible explanations, these mechanisms were not directly evaluated. Therefore, the association between cloprostenol treatment and lower early abortion rate should be considered a field observation that requires confirmation in future studies.

Several limitations of the present study should be acknowledged. Serum progesterone concentrations were not measured, and ovarian ultrasonography was not performed before treatment; therefore, residual luteal activity, lactational ovulation, or other ovarian structures could not be confirmed. Finally, the study evaluated early reproductive indicators up to 42 days of gestation and did not include farrowing rate, total born piglets, or subsequent litter performance. For this reason, the findings should not be extrapolated to overall reproductive performance without further confirmation.

In conclusion, under the commercial conditions of this field study, pre‐weaning administration of cloprostenol was associated with a shorter WFSI, a higher proportion of sows showing oestrus within 7 days after weaning, and a lower early abortion rate, without significant effects on pregnancy rate or early endometritis incidence. Further studies combining reproductive performance data with endocrine profiling, ovarian ultrasonography and farrowing outcomes are needed to confirm the biological mechanisms and long‐term reproductive relevance of these findings.

## Author Contributions

Experimental design: S.C. and J.G. Experimental work: S.C. Data analysis: J.G. Writing of the manuscript: S.C. and J.G. All authors have revised and approved the final version of the manuscript.

## Funding

University of Murcia and CEFU S.A. collaboration project (34071). Project 23,031/GERM/25 supported by FSRM/10.13039/100007801.

## Conflicts of Interest

The authors declare no conflicts of interest.

## Data Availability

The data that support the findings of this study are available from the corresponding author upon reasonable request.
